# Mechanical Harvesting Effectively Controls Young *Typha* spp. Invasion and Unmanned Aerial Vehicle Data Enhances Post-treatment Monitoring

**DOI:** 10.3389/fpls.2017.00619

**Published:** 2017-04-25

**Authors:** Shane C. Lishawa, Brendan D. Carson, Jodi S. Brandt, Jason M. Tallant, Nicholas J. Reo, Dennis A. Albert, Andrew M. Monks, Joseph M. Lautenbach, Eric Clark

**Affiliations:** ^1^Institute of Environmental Sustainability, Loyola University ChicagoChicago, IL, USA; ^2^Human Environment Systems Center, Boise State UniversityBoise, ID, USA; ^3^University of Michigan Biological StationPellston, MI, USA; ^4^Native American and Environmental Studies, Dartmouth CollegeHanover, NH, USA; ^5^Department of Horticulture, Oregon State UniversityCorvallis, OR, USA; ^6^Inland Fish and Wildlife Department, Sault Ste. Marie Tribe of Chippewa IndiansSault Ste. Marie, MI, USA

**Keywords:** Great Lakes, wetlands, biological invasions, ecological restoration, early detection and rapid response, UAV remote sensing, restoration monitoring

## Abstract

The ecological impacts of invasive plants increase dramatically with time since invasion. Targeting young populations for treatment is therefore an economically and ecologically effective management approach, especially when linked to post-treatment monitoring to evaluate the efficacy of management. However, collecting detailed field-based post-treatment data is prohibitively expensive, typically resulting in inadequate documentation of the ecological effects of invasive plant management. Alternative approaches, such as remote detection with unmanned aerial vehicles (UAV), provide an opportunity to advance the science and practice of restoration ecology. In this study, we sought to determine the plant community response to different mechanical removal treatments to a dominant invasive wetland macrophyte (*Typha* spp.) along an age-gradient within a Great Lakes coastal wetland. We assessed the post-treatment responses with both intensive field vegetation and UAV data. Prior to treatment, the oldest *Typha* stands had the lowest plant diversity, lowest native sedge (*Carex* spp.) cover, and the greatest *Typha* cover. Following treatment, plots that were mechanically harvested below the surface of the water differed from unharvested control and above-water harvested plots for several plant community measures, including lower *Typha* dominance, lower native plant cover, and greater floating and submerged aquatic species cover. Repeated-measures analysis revealed that above-water cutting increased plant diversity and aquatic species cover across all ages, and maintained native *Carex* spp. cover in the youngest portions of *Typha* stands. UAV data revealed significant post-treatment differences in normalized difference vegetation index (NDVI) scores, blue band reflectance, and vegetation height, and these remotely collected measures corresponded to field observations. Our findings suggest that both mechanically harvesting the above-water biomass of young *Typha* stands and harvesting older stands below-water will promote overall native community resilience, and increase the abundance of the floating and submerged aquatic plant guilds, which are the most vulnerable to invasions by large macrophytes. UAV's provided fast and spatially expansive data compared to field monitoring, and effectively measured plant community structural responses to different treatments. Study results suggest pairing UAV flights with targeted field data collection to maximize the quality of post-restoration vegetation monitoring.

## Introduction

The ecological impacts of invasive plants tend to compound temporally (Strayer et al., 2006; Mitchell et al., 2011; Lishawa et al., 2013; Simberloff et al., 2013), resulting in biodiversity losses, plant community restructuring, and eventually the alteration of biogeochemical cycling with time since introduction (Suding et al., 2004; Suding and Hobbs, 2009; Zedler, 2009). Theory suggests that long-invaded ecosystems can enter *alternative stable states* (Beisner et al., 2003) outside of their historical range of variability, and become increasingly difficult to restore (Suding et al., 2004). The shorter the duration of time between invasion and when control activities occur, the more likely the invasion will be stemmed, preventing an ecosystem from reaching an alternative stable state. Evidence from invasive plant eradication efforts support this perspective: as invasions progress in age and size, eradication becomes less likely and more expensive (Rejmánek and Pitcairn, 2002). Thus, early detection and rapid response (EDRR) to invasive species is viewed as the most economically efficient and ecologically effective approach (Hobbs and Humphries, 1995; Vander Zanden et al., 2010; Simberloff et al., 2013). Federal and state invasive plant management programs increasingly reflect this perspective (Westbrooks, 2004). Despite the clear ecological and economic advantages of controlling invasive plants soon following invasion, in practice most control efforts do not begin until invaders have achieved high levels of dominance. Therefore, it is not surprising that large-scale invasive plant control tends to be an expensive, labor and chemical intensive process, which often results in unsatisfactory outcomes (Martin and Blossey, 2013; Simberloff, 2014).

While a quick response to newly detected and small-scale invasions may result in better control of the target species (Rejmánek and Pitcairn, 2002), there are limited examples of invasive plant management occurring on new populations and there is sparse research documenting the relative effectiveness of invasive plant control efforts targeting new invasions. This is in part because managers tend not to be rewarded for publishing (Simberloff, 2009) and because few coordinated and well-funded EDRR systems exist (Simberloff, 2014). Further, because newly invaded ecosystems are more likely to continue harboring diverse and high quality plant communities, the potential for widely-utilized management techniques to result in collateral damage to native flora and fauna must be recognized. For instance, herbicide application is the most commonly used approach to control invasive wetland macrophytes such as *Phragmites australis* and *Typha* × *glauca* in eastern North America (Homan et al., 2003; Linz and Homan, 2011; Martin and Blossey, 2013) and is the most commonly studied invasive plant control technique (Kettenring and Adams, 2011). Herbicide use can result in unintended ecosystem impacts such as directly killing non-target species (Matarczyk et al., 2002), altering algal communities (Saxton et al., 2011), and increasing nutrient availability (Linz and Homan, 2011; Lawrence et al., 2016b). Mechanical control of invasive wetland macrophytes has the potential to increase native plant diversity (Lishawa et al., 2015), while avoiding some of the unintended consequences associated with chemical control.

Here, we sought to test mechanical invasive plant control methods in a high quality northern Great Lakes coastal wetland with a young and expanding population of a clonal invasive macrophyte, *Typha* × *glauca* (hereafter *Typha*). Invasive *Typha* has become increasingly wide-spread and dominant in the northern Great Lakes since the late 1990's associated with a prolonged period of low water levels (Lishawa et al., 2010). Most climate change models predict long-term reductions in Great Lakes water levels over the next century (Angel and Kunkel, 2010) creating conditions more favorable for *Typha*. Invasive *Typha* dominance has detrimental impacts on native flora and fauna including reducing plant diversity (Frieswyk and Zedler, 2007; Wilcox et al., 2008), altering plant community structure (Lishawa et al., 2010), and reducing macroinvertebrate abundance (Lawrence et al., 2016a) in Great Lakes coastal wetlands. As stands of *Typha* age, plant diversity decreases and litter accumulates (Mitchell et al., 2011; Lishawa et al., 2013), resulting in reduced seed bank recruitment and native seed germination (Frieswyk and Zedler, 2006; Lishawa et al., 2015). In highly dominant stands, above-water mechanical removal of *Typha* has been demonstrated to increase native plant diversity and reduce *Typha* dominance (Lishawa et al., 2015) and cutting stems below-water can effectively kill *Typha* (Apfelbaum, 1985).

Increased funding and emphasis on EDRR would almost certainly improve the efficacy of invasive plant management. However, even with a robust system in place it is critical that detailed monitoring of post-treatment responses be conducted in order to evaluate the effectiveness of EDRR intervention (Simberloff, 2009). Monitoring is an often-overlooked or inadequately implemented aspect of invasive species management because of the expense of conducting vegetation surveys, the focus of granting agencies on acreage as the principal metric for evaluating restoration success, and in many cases monitoring and research are explicitly excluded from funding opportunities.

Unmanned aerial vehicles (UAVs) have the potential to deliver the crucial data needed to monitor the response of vegetation to restoration. Recent advances in UAV data collection, processing, and analysis techniques have already begun to drive progress in plant ecology. For example, in inaccessible wetlands UAVs have been used to remotely detect invasive species (Hill et al., 2017) and characterize wetland bird habitat quality (Chabot et al., 2014). In addition, data from UAVs have been used to conduct rapid assessment in remote locations, including fine-scale vegetation structure (Fraser et al., 2016), and biomass assessments (Messinger et al., 2016). Despite their excellent potential (Knoth et al., 2013), the utility of UAVs for monitoring invasive species management and restoration responses has not been extensively assessed.

The principal objectives of our study were 2-fold: to evaluate the effectiveness of non-chemical invasive plant control methods on a burgeoning population of a clonal invasive macrophyte, *Typha* × *glauca*, in a high quality Great Lakes coastal wetland; and to evaluate the efficacy of using UAV collected data to monitor post-treatment vegetation responses. We established experimental above and below-water harvest treatments in five nascent *Typha* stands and extended our treatments beyond the area dominated by *Typha* into native-plant dominated wetland to evaluate the resilience of native flora to mechanical treatment. We collected plant community data to characterize plant composition and dominance. Secondarily, we sought to explore the relationship between *Typha* stand-age and a suite of plant community characteristics. Finally, we evaluated UAV-collected data and compared those data with similar field-measured metrics. We tested the hypotheses that: (1a) portions of the study wetland occupied by older *Typha* would be more impacted by the invasive plant and (1b) more resistant to restoration efforts than areas with younger *Typha*; (2) above-water harvesting would reduce *Typha* dominance, alter the physical structure of the vegetation, and increase native species diversity, (3) below-water harvesting would more effectively reduce *Typha* dominance, but do so at the cost of also reducing native species diversity, and (4) the effects our experimental manipulation has on vegetation structural composition will be similarly detectable through both traditional on-the-ground surveys and remote sensing analysis.

## Materials and methods

### Study site

**Figure 1:**
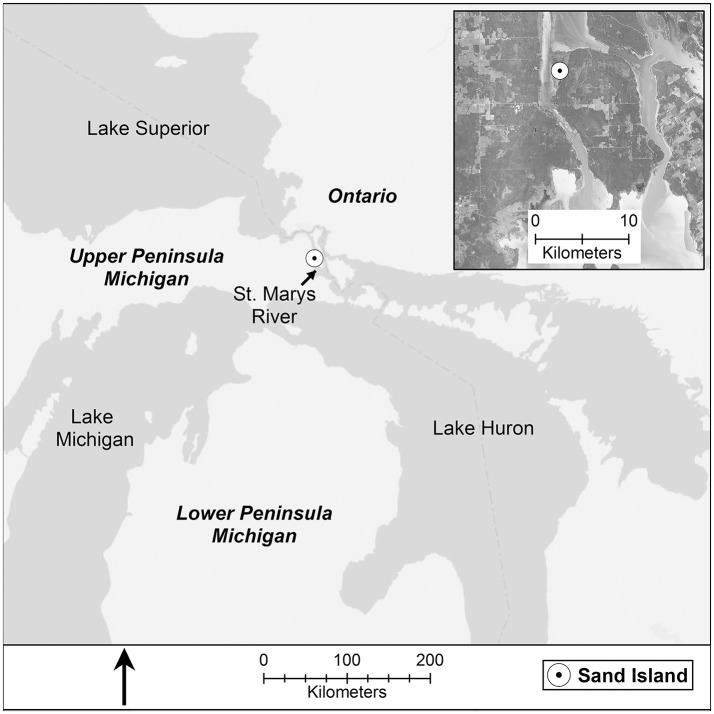
**Study region, the St. Marys River, the connecting channel between Lake Superior to the north and Lake Huron to the south**.

We conducted this study in an 80 hectare *connecting channel protected embayment* (Albert et al., 2005) Great Lakes coastal wetland on Sand Island, a peninsula at the northwest corner of Neebish Island, in the St. Marys River, Michigan, USA (N 46.31362°, W 84.19725°; Figure 1). The St. Marys River is the connecting channel between Lake Superior and Lake Huron and is the border between Michigan (USA) and Ontario (Canada) for its full 120 km length. The wetland soil profile of Sand Island marsh typically consists of a shallow organic layer (~10 cm) grading into a band of sandy silt (10–20 cm) overlaying lacustrine clay.

### Experimental evaluation of EDRR strategies

#### Study design

**Figure 2:**
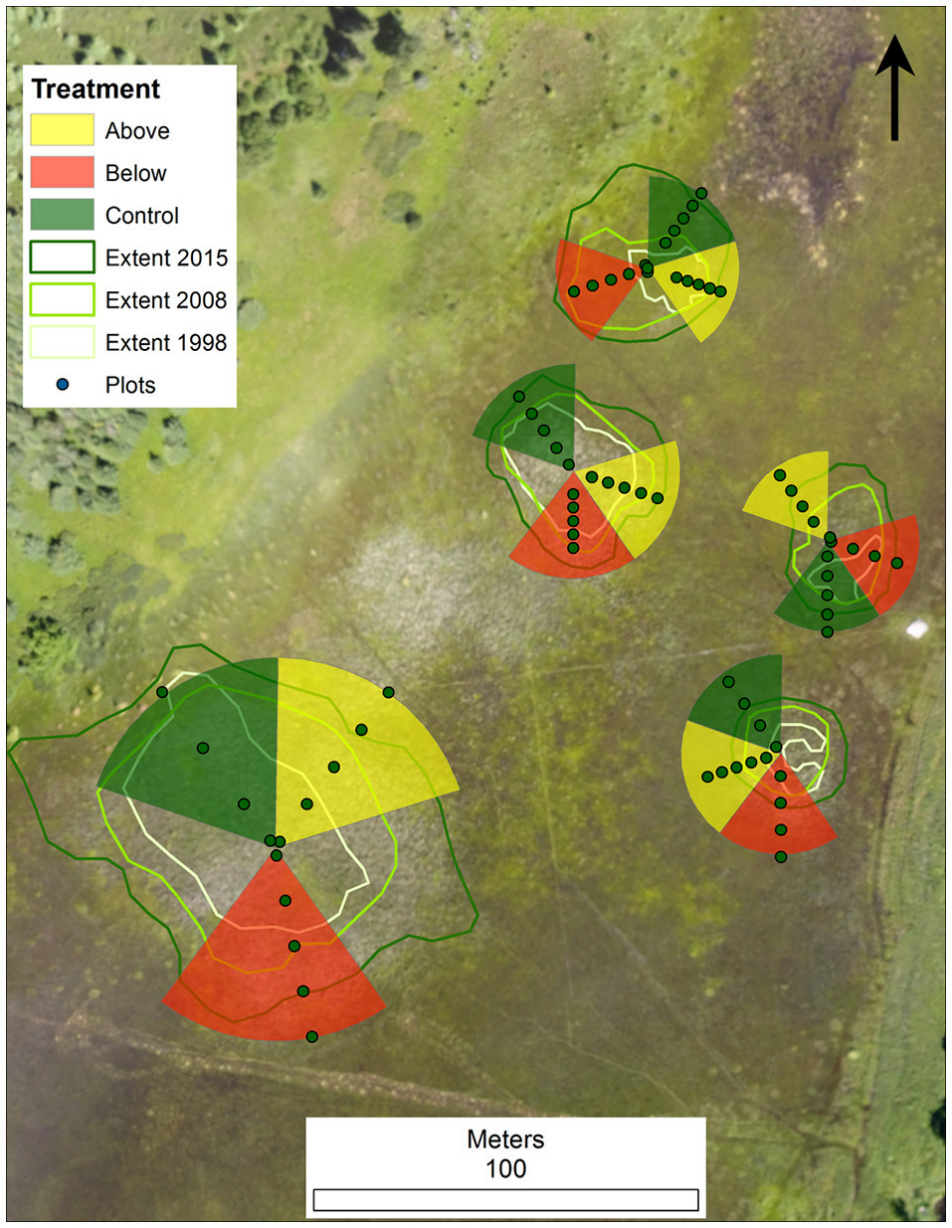
**Plot layout at Sand Island, MI illustrating five isolated ***Typha*** stands, completely randomized block design, vegetation sampling transects, vegetation subplots, and the extent of ***Typha*** at three points in time (1998, 2008, and 2015)**.

Using publically available aerial imagery data from the US Department of Agriculture's Farm Service Agency National Agricultural Inventory Program (NAIP), we identified 5 circular *Typha* stands at the north end of the wetland, and confirmed their size and invasive *Typha* dominance with ground-truthing. The stands were isolated from each other but close in proximity (between 20 and 50 m) and were growing in a matrix of native sedge (*Carex lasiocarpa* and *C. aquatilis*) and hardstem bulrush (*Schoenoplectus acutus*) dominated wetland. Stands ranged in size from 2,400–7,800 square meters in 2015 (Figure 2).

We used a completely randomized block design that treated individual stands as a block, divided each block into 5 equally sized wedges, and randomly assigned each wedge to one of three treatments: above-water biomass harvest, below-water biomass harvest, or control. Two additional above-water wedges (beyond one per block) were initially assigned in order to allow for repeated treatments to be conducted in year-2. To avoid unbalanced data, one wedge of each treatment per block was randomly selected for all post-treatment comparisons (Figure 2). We identified the geographic center of each stand by running two 100 m fiberglass measuring tapes along north-south and east-west bearings. To evaluate vegetation responses along a gradient from *Typha* dominated to native plant dominated wetland, treatment wedges were extended from stand center to 10 m beyond the *Typha* dominated margin of the stand. We established above-water harvest treatments on August 20–30, 2015 using a low-ground pressure wetland plant harvester (Loglogic Softrak with Cut and Collect system, Devon England, U.K.) and conducted below water treatments using aquatic weed-whackers (Weeders Digest LLC, New Hope, MN, U.S.A.) followed by hand biomass removal.

#### Field data collection

On August 19–27, 2015, prior to treatment implementation, we established a series of five 1 × 1 m subplots, equally spaced along transects bisecting the middle of each wedge and extending from block center to 10 m beyond the edge of the core of the *Typha* stand. Thus, each subplot fell along a gradient from the interior to exterior of the *Typha* stand. We collected vegetation data by assigning aerial cover values (<1–100%) for total vegetative cover, detritus (below water litter), standing dead (above water) litter, and for each plant species. Additionally, we calculated aboveground *Typha* biomass by measuring the height of each individual stem and using a height-to-dry biomass allometric equation (*g* = 0.5265e1.751^*^height (m), *r*^2^ = 0.81; Lishawa et al., 2015). One year following treatment implementation, on August 18–20, 2016, we resampled vegetation subplots using the same methods.

#### Aging *Typha* stands

We used two high resolution (1 ft pixel), high quality historical aerial photographs from 1998 and 2008 (MDNR, 2001; USGS, 2008) along with field collected data from 2015 to post-dict the invasion history of *Typha* within the study site following the methods of Lishawa et al. (2013). We determined the extent of *Typha* in 2015 through field observation; created a shapefile demarcating the stand boundaries; and evaluated the two selected images counter-chronologically and created *Typha* extent polygons from each. Individual vegetation plots were assigned to one of four stand-age classes representing the first documented date when *Typha* dominated (subdominant, 2015, 2008, 1998); the subdominant category had scattered *Typha* stems (<5/m^2^).

### UAV materials and methods

#### Aerial photography collection

We collected ultra-high resolution (0.8 cm pixel) imagery using an unmanned aerial vehicle (eBee, senseFly SA, Chesaux-Lausanne Switzerland), equipped with color (RGB) and near infrared (NIR) cameras (RGB G9X and S110 NIR, Canon Inc. Melville, NY U.S.A.) on August 3, 2016, 1-year after treatments were implemented. Payload was limited to one camera per flight, so RGB and NIR were flown separately, within the same hour and same condition. Flight planning software (eMotion 2 Version 2.3.11, senseFly SA, Chesaux-Lausanne Switzerland) was used to automate image collection and replicate collection areas between camera flights. We used an 80% overlap of images, which is required to accurately match images in Agisoft Photoscan structure from motion (SfM) software (Agisoft PhotoScan; Agisoft LLC, St. Petersburg Russia).

#### Image post processing and georeferencing

All collected images were processed in Agisoft Photoscan SfM software, which provides a workflow for creating ultra-high resolution mosaics using UAV collected images. The software uses camera location, as determined by the integrated GNSS receiver, and matches pixels between images to align the many pictures taken by the UAV into orthomosiacs. Georeferencing of imagery was performed in conjunction with the creation of a site level orthomosiac using the camera locations.

#### Normalized difference vegetation index

We calculated raw normalized difference vegetation index [raw NDVI sensu (Nebiker et al., 2008), hereafter NDVI) scores using the digital number (DN) values and the NDVI function in ArcGIS, which uses the following equation (NDVI = (IR-R)/(IR + R)]. We classified three land cover categories based on NDVI: (1) water (NDVI < 0), (2) brown vegetation (NDVI between 0 and 0.28), and (3) green vegetation (NDVI > 0.28; Table 1).

**Table 1:** **Unmanned aerial vehicle collected data and equivalent field measure**.

**UAV measure**	**Calculation**	**What it represents**	**Equivalent field measure**
NDVI	(NIR-Red)/(NIR + Red)	Photosynthetic vegetation	Total vegetation cover
Green vegetation cover	NDVI > 0.28	Green vegetation	Total vegetation cover
Brown vegetation cover	NDVI > 0 < 0.28	Non-photosynthetic vegetation/Litter	Total detritus values
Open water	NDVI < 0	Open water	Unvegetated cover
Blue band reflectance	Raw DN (RGB image; Blue band)	Alternative open water	Unvegetated cover
Surface height	μ corrected digital surface model pixel value by treatment plot	Vegetation canopy height	No equivalent
Surface height range	Max pixel elevation—min pixel elevation by treatment plot	Variability of canopy surface	No equivalent
Surface height standard deviation	$\sqrt{\frac{\sum {\left|x-\bar{x}\right|}^{2}}{n}}$	Complexity of canopy surface	No equivalent
Green tissue height	Derived from NDVI and DSM	Living plant canopy height	*Typha* height measures
Brown tissue height	Derived from NDVI and DSM	Standing dead tissue canopy height	Litter height

#### Vegetation structure

Using SfM software, we produced surface elevation models by creating a point cloud using photogrammetry of overlapping, georeferenced images (Westoby et al., 2012). We corrected elevation data in order to compare with field-measured vegetation height data by subtracting the average elevation of pixels identified as water (i.e., elevation of water) and adding the average field measured water depth; the resulting numbers (surface height) represent the total height of vegetation above the sediment surface.

#### Plot-level summaries of UAV-derived measures

We used the zonal statistics tool in ArcGIS to summarize the various output rasters by treatment plots (NDVI, land cover category, surface elevation, and land cover class elevation). Secondly, we calculated the range and standard deviation of surface elevation heights within each plot and for each land cover category within each plot (Table 1).

### Statistical analysis

We sought to statistically test: (1) the pre-treatment effects of stand age, stand, and subplot on a set of field-derived vegetation response variables that describe *Typha* and native plant dominance (*Typha* biomass, *Typha* cover, H′, species richness, vegetative cover, standing dead cover, detritus cover, *Carex* spp. cover, and floating and submerged aquatic species cover); (2) the effects of treatments (above water harvest (AW), below water harvest (BW), control) on the same field-derived vegetation response variables; and (3) the effects of treatments on remotely detected land cover classes, vegetation structure, and reflectance values (percent green vegetation cover, percent brown vegetation cover, percent water cover, surface elevation, elevation range, NDVI, blue band reflectance). Pre-treatment vegetation data were evaluated using analysis of variance (ANOVA) and linear mixed effects modeling (LME) to determine the effects of age, stand, and subplot location relative to the stand center (i.e., subplot). Tukey's HSD tests were used to evaluate pairwise differences among groups. We evaluated the effects of restoration treatments on response variables and remotely detected vegetation structure and reflectance values (post-treatment effects) using LME. *Typha* stand was used as a source of random effects in all LME models, which allowed us to account for non-independence among these measurements. We used indicator species analysis (Dufrene and Legendre, 1997) to find correspondence between individual species and age and treatment (above water, below water, control) from pre-treatment and post-treatment vegetation data, respectively. Indicator values of plant species were tested via Monte-Carlo simulation using 1,000 permutations. To meet model assumptions of residual normality, we transformed response data using either log or arcsin-square root transformations, when necessary. All statistical analyses were performed in R 2.15.0 (R Core Team, 2013), using the lme4 package to analyze LME models (Bates et al., 2015).

## Results

### Experimental evaluation of restoration strategies

#### Pre-treatment data

Data collected in August 2015, and averaged across all stands, revealed that *Typha* stand-age was strongly associated with a range of measured environmental and plant community variables (Tables 2, 3). Particularly, the oldest age class (1998) differed from the subdominant and youngest *Typha* classes in measures of *Typha* dominance (*Typha* biomass, *Typha* cover, detritus cover; all *P* < 0.01) and native plant community composition [*Carex* spp. cover, Shannon diversity (H′), species richness; all *P* < 0.05; Table 3]. Similarly, the same measures of *Typha* dominance and native plant community composition tended to vary with pre-treatment subplot location, which reflects the relative distance into the center of a *Typha* stand (*A* = outside *Typha* dominated stand → *E* = near stand center; Figure 2; Tables 2, 4). The two most exterior subplots (A, B) differed from each other only in *Typha* biomass (*P* < 0.01) and *Carex* spp. cover (*P* < 0.01), whereas both subplots differed from the three most interior subplots (C, D, E) in nearly all other measured values. The three most interior subplots did not differ in any measured value (Table 4). Indicator species analysis revealed two species were correlated with individual stand-age categories, *Carex lacustris* correlated with the oldest age class (1998; IV, 39.3%; *P* < 0.05) and *C. utriculata* was associated with the subdominant group (IV, 34.3%; *P* < 0.05), where indicator values (IV) represent the percentage of perfect indication for each group.

**Table 2:** **Results of statistical tests (ANOVA) evaluating the independent effects of age, stand, and subplot (proximity to stand center) on plant and environmental conditions in 2015, prior to treatment implementation**.

**Variable**	**Factor**	***df***	***F***	***P***
*Typha* biomass (g/m^2^)	Age	3	11.24	<0.0001^***^
	Stand	4	2.15	0.0794•
	Subplot	4	30.59	<0.0001^***^
*Typha* cover (%)	Age	3	8.63	0.0004^***^
	Stand	4	0.38	0.8200
	Subplot	4	12.58	<0.0001^***^
Standing dead (%)	Age	3	18.48	<0.0001^***^
	Stand	4	5.47	0.0016^**^
	Subplot	4	8.91	<0.0001^***^
Detritus (%)	Age	3	3.58	0.0163^*^
	Stand	4	39.6	<0.0001^***^
	Subplot	4	5.62	0.0004^***^
Total vegetation cover (%)	Age	3	4.82	0.0035^**^
	Stand	4	7.56	0.0002^***^
	Subplot	4	0.68	0.6100
*Carex* spp. cover (%)	Age	3	10.81	<0.0001^***^
	Stand	4	12.27	<0.0001^***^
	Subplot	4	8.15	<0.0001^***^
*Aquatic* spp. cover (%)	Age	3	1.35	0.263
	Stand	4	3.93	0.0049^**^
	Subplot	4	4.49	0.002^**^
H′	Age	3	1.94	0.127
	Stand	4	6.32	0.0001^***^
	Subplot	4	4.49	0.0022^**^
Species richness	Age	3	2.05	0.112
	Stand	4	6.60	0.0008^***^
	Subplot	4	3.66	0.0078^**^

• *P < 0.10*.

* *P < 0.05*.

** *P < 0.01*.

*** *P < 0.001*.

**Table 3:** **Pairwise comparison of variable values by age classes determined by LME model (with ***Typha*** stand as a source of random effects) and Tukey ***post-hoc*** tests**.

**Variable**	**1998: Subdominant**	**2008: Subdominant**	**2015: Subdominant**	**1998: 2008**	**1998: 2015**	**2008: 2015**
	*P*	*P*	*P*	*P*	*P*	*P*
*Typha* biomass (g/m^2^)	^***^	^***^	^*^	0.51	^**^	0.29
*Typha* (%)	^***^	^*^	0.93	0.6	^**^	•
Standing dead (%)	^***^	^*^	0.37	0.56	0.19	0.85
Detritus (%)	^***^	^***^	0.21	0.13	^***^	0.29
Total vegetation (%)	^**^	0.70	0.27	^*^	0.46	0.83
*Carex* spp. (%)	^***^	^***^	0.85	0.97	^***^	^**^
*Aquatic* spp. (%)	•	0.43	0.46	0.77	0.87	0.99
H′	^*^	0.20	0.99	0.69	^*^	0.42
Species richness	^*^	0.18	0.97	0.57	^*^	0.49

• *P < 0.10*.

* *P < 0.05*.

** *P < 0.01*.

*** *P < 0.001*.

**Table 4:** **Pairwise comparison by subplot determined by LME model (with ***Typha*** stand as a source of random effects) and Tukey ***post-hoc*** tests**.

**Variable**	**A:B**	**A:C**	**A:D**	**A:E**	**B:C**	**B:D**	**B:E**	**C:D**	**C:E**	**D:E**
	*P*	*P*	*P*	*P*	*P*	*P*	*P*	*P*	*P*	*P*
*Typha* biomass (g/m^2^)	^**^	^***^	^***^	^***^	^***^	^***^	^***^	0.96	0.86	0.99
*Typha* cover (%)	•	^***^	^***^	^***^	^**^	^**^	^***^	0.99	0.98	0.99
Standing dead (%)	0.5	^***^	^***^	^***^	^*^	^*^	•	0.99	0.99	0.99
Detritus (%)	0.89	^***^	^***^	^***^	^*^	^***^	^***^	0.7	0.26	0.95
*Carex* cover (%)	^**^	^***^	^***^	^***^	^***^	^***^	^***^	0.96	0.86	0.99
H′	0.92	•	^**^	^*^	^**^	^***^	^**^	0.96	0.99	0.99
Species richness	0.99	^*^	^*^	^**^	^*^	^**^	^**^	0.99	0.99	0.99

*As subplot enumeration increases (A -> E) location approached the center of the Typha stand. Only those variables which had significant subplot effect are shown*.

• *P < 0.10*.

* *P < 0.05*.

** *P < 0.01*.

*** *P < 0.001*.

We found that with the exception of *Typha* biomass and *Typha* cover, environmental and plant community measures differed by *Typha* stand (Table 2), supporting the need to use stand as a random effect in linear mixed effects (LME) models.

#### Treatment response

**Figure 3:**
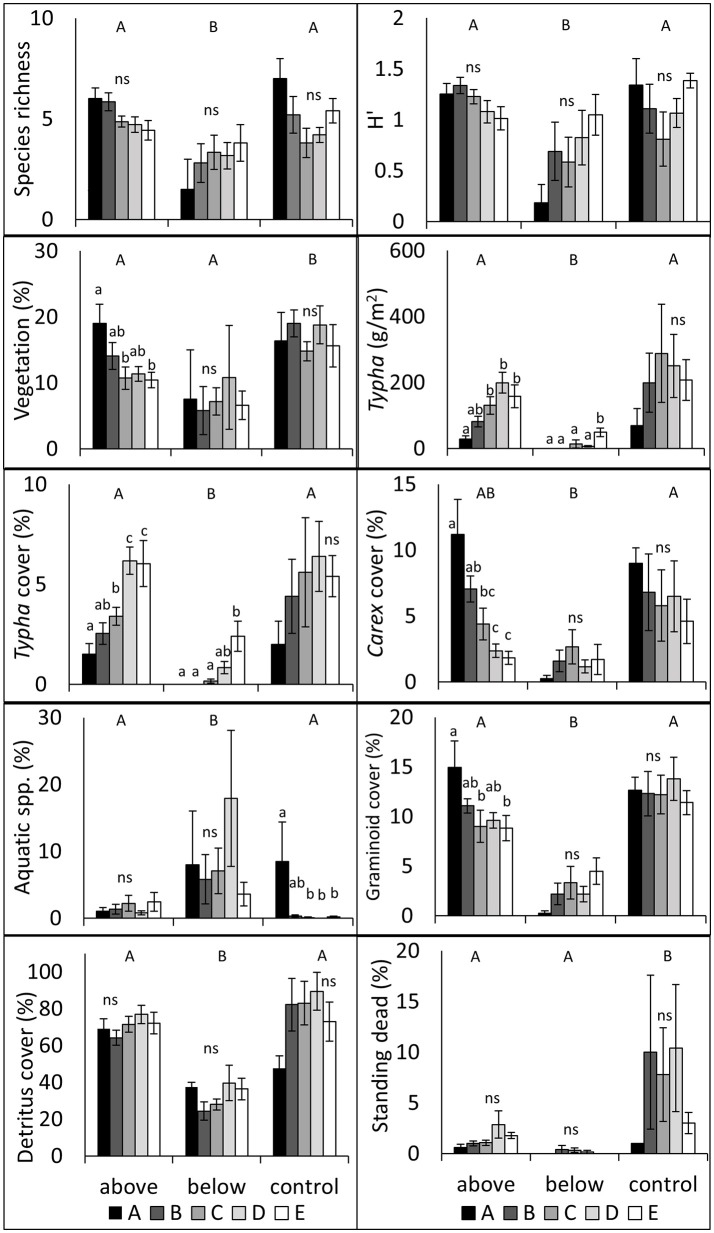
**Vegetation responses by treatment and subplot ± SE in 2016, 1-year following treatment implementation**. Capital letters denote significant treatment contrasts; lowercase letters denote within treatment subplot contrasts; ns denotes no significant differences (*P* > 0.05) between subplots. Subplots approach the center of each stand with alphabetically increasing enumeration.

Data collected 1-year following treatment (August 2016) illustrated treatment effects and differences between subplots (Figure 3). We found that BW treatment responses differed from AW and control treatments in almost all measured variables; species richness, H′, *Typha* biomass, *Typha* cover, graminoid cover, and total detritus cover were all lower, and floating and submerged aquatic spp. cover was higher in BW treatments (all *P* < 0.05). AW only differed from control in two measures: total vegetation cover and standing dead cover, both of which were higher in the control treatments (all *P* < 0.05). Data also varied within treatments and between subplots, particularly within the AW treatment; notably, *Carex* spp. cover decreased and *Typha* cover and biomass increased as subplot locations approached the stand center.

Repeated measures LME modeling further illuminated the effects of stand-age, year, and their interactions on measured variables (Table 5). *Typha* cover, standing dead, and total vegetation cover all declined between year 1 and year 2 in all treatments (all *P* < 0.05). The BW treatment decreased *Typha* biomass (Year: *F* = 24.75 *P* < 0.0001) and total detritus (Year: *F* = 16.74, *P* < 0.01), *Carex* spp. cover (*F* = 25.18, *P* < 0.0001), plant diversity (H′; *F* = 6.41, *P* < 0.05), and species richness (*F* = 9.88, *P* < 0.001). AW increased total detritus (Year: *F* = 17.31, *P* < 0.001), H′ (*F* = 5.44, *P* < 0.05) and had no effect on richness. Aquatic species cover decreased between pre-treatment and post-treatment in the control plots, but increased in the AW plots (*F* = 5.75, *P* < 0.01).

**Table 5:** **Results of linear mixed effects model (with ***Typha*** stand as a source of random effects) evaluating the effects of stand-age and Year (pre-treatment in year-1) on plant and environmental conditions**.

**Variable**	**Factor**	**Control**				**Below water**			**Above water**		
		**Effect**				**Effect**			**Effect**		
		***df***	***F***	***P***	**±**	***F***	***P***	**±**	***F***	***P***	**±**
*Typha*	Stand-age	3	14.00	^***^	+	1.53	.		11.95	^***^	+
biomass	Year	1	0.31	.		24.75	^***^	–	0.00	.	
(g/m^2^)	Stand-age^*^Year	3	1.69	.		0.35	.		1.01	.	
*Typha* cover	Stand-age	3	10.78	^**^	+	1.32	.		11.37	^***^	+
(%)	Year	1	13.32	^**^	–	44.86	^***^	–	10.05	^**^	–
	Stand-age^*^Year	3	1.89	.		0.32	.		2.00	.	
Standing dead	Stand-age	3	0.85	•	+	0.98	.		4.88	^**^	+
(%)	Year	1	8.93	^*^	–	41.34	^***^	–	54.31	^***^	–
	Stand-age^*^Year	3	0.21	.		1.47	.		3.50	^*^	
Total detritus	Stand-age	3	3.80	^*^	+	1.76	.		6.02	^**^	+
(%)	Year	1	0.29	.		16.74	^**^	–	17.31	^***^	+
	Stand-age^*^Year	3	3.11	^*^		2.20	.		1.62	.	
Total vegetation cover (%)	Stand-age	3	1.05	.		0.73	.		6.44	^***^	–
	Year	1	6.39	^*^	–	46.71	^***^	–	169.95	^***^	–
	Stand-age^*^Year	3	0.84	.		1.99	.		0.61	.	
*Carex* spp.	Stand-age	3	2.19	.		2.71	•	–	10.62	^***^	–
cover (%)	Year	1	0.33	.		25.18	^***^	–	42.79	^***^	–
	Stand-age^*^Year	3	0.04	.		1.63	.		4.10	^**^	
*Aquatic* spp.	Stand-age	3	1.45	.		0.13	.		1.24	.	
cover (%)	Year	1	5.91	^*^	–	2.94	•	+	5.75	^**^	+
	Stand-age^*^Year	3	1.16	.		1.40	.		1.87	.	
H′	Stand-age	3	0.54	.		0.73	.		2.26	•	–
	Year	1	0.54	.		6.41	^*^	–	5.44	^*^	+
	Stand-age^*^Year	3	0.57	.		1.69	.		0.86	.	
Species richness	Stand-age	3	0.41	.		0.93	.		4.13	^**^	–
	Year	1	0.36	.		9.88	^**^	–	0.24	.	
	Stand-age^*^Year	3	1.05	.		1.54	.		0.44	.	

*Directionality of significant effects indicated by a positive or negative sign*.

. *P > 0.10*.

• *P < 0.10*.

* *P < 0.05*.

** *P < 0.01*.

*** *P < 0.001*.

Indicator species analysis revealed that the submerged aquatic species *Utricularia intermedia* was significantly associated with the BW treatment with an IV of 83.3% of perfect indication, while *C. lacustris* and *Fraxinus* spp. were associated with the control treatment 55.8 and 36.1% IV, respectively (all *P* < 0.05).

### Evaluation of UAV data for post-restoration monitoring

#### UAV data and comparison with field data

**Figure 4:**
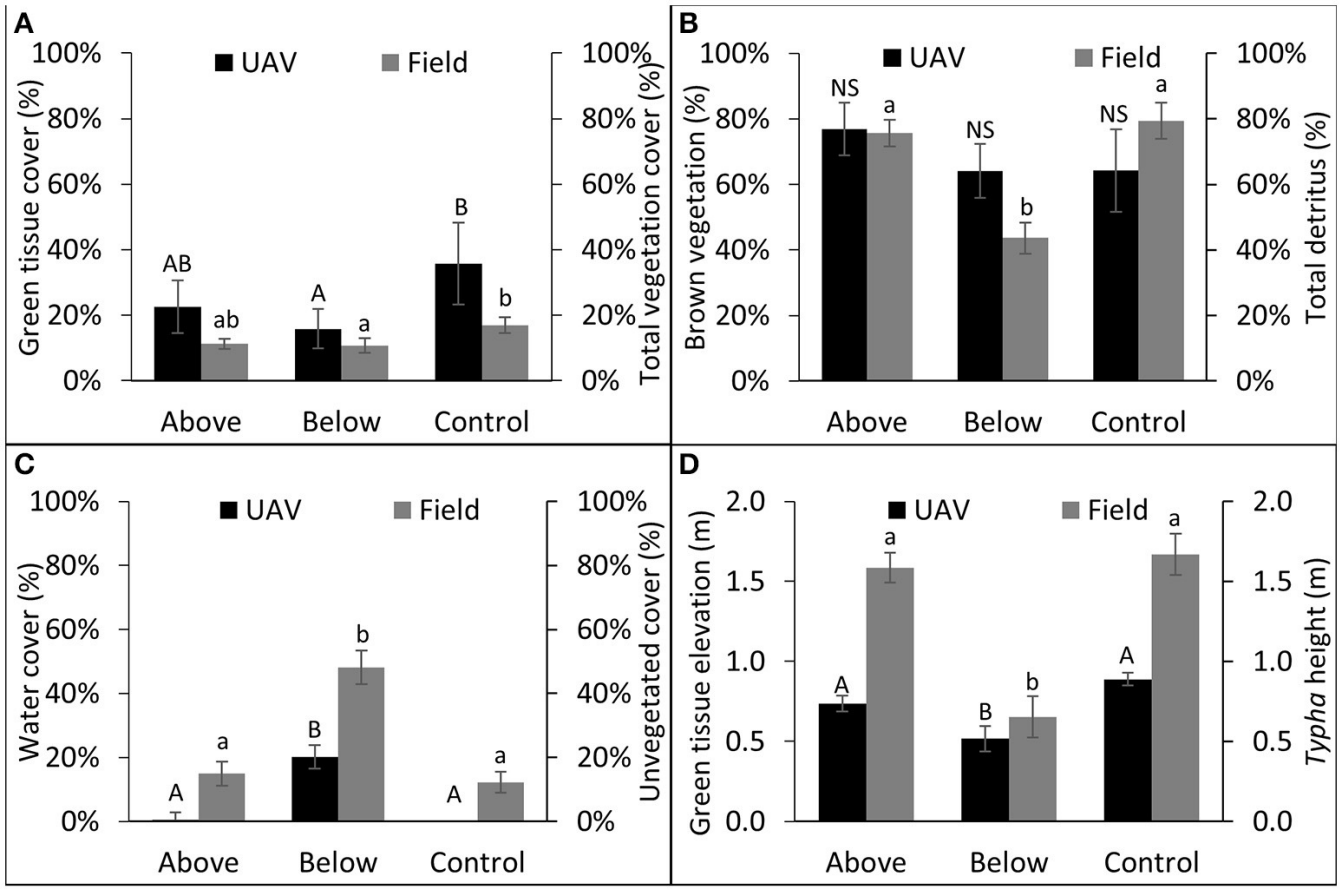
**Comparison between UAV collected data and field collected data; UAV variable on left axes and comparable field collected data on right axes**. Letter differences denote significant treatment contrasts, UAV data represented by capital letters, field data represented by lowercase letters. NS denotes no significant differences between treatments (*P* > 0.05). **(A)** UAV green tissue cover (%) (area with NDVI value > 0.28/total area); **(B)** UAV brown tissue cover (%) (area with NDVI values between 0.0 and 0.28/total area); **(C)** UAV water cover (%) (area with NDVI value > 0.0/total area); **(D)** green tissue height (corrected using water average water elevation).

UAV-derived vegetation data illustrated similar treatment effects as field measured data (Figure 4). Control plots had greater total vegetation cover values (UAV: μ = 0.36 ± 0.13; Field: μ = 0.17 ± 0.02) than BW plots (UAV: μ = 0.17 ± 0.08; Field: μ = 0.11 ± 0.02; Figure 4A), and values did not differ from AW plots (UAV: μ = 0.23 ± 0.08; Field: μ = 0.11 ± 0.02). Mean NDVI derived brown vegetation cover values compared closely with field measured total detritus values in the AW (UAV:μ = 0.77 ± 0.08; Field: μ = 0.76 ± 0.04) and control plots (UAV:μ = 0.64 ± 0.13; Field: μ = 0.79 ± 0.05), but differed substantially from the BW plots (UAV:μ = 0.64 ± 0.08; Field: μ = 0.44 ± 0.05). In the field data AW and control plots had significantly greater detritus than in BW plots (*P* < 0.05), whereas UAV data did not differ significantly between treatments. The percentage of each plot covered with open water, and the equivalent field measure (percent unvegetated cover), varied between the UAV and the field data, but in both cases the BW plots had significantly greater open water cover than the AW or control plots (Figure 4C). Finally, the average green tissue height values, derived from NDVI scores and the digital surface model, varied from field measured *Typha* height values in both the AW and control plots, though the BW treatment was significantly lower than AW or control treatments in both cases (*P* < 0.05; Figure 4D).

#### Treatment impacts as measured by the UAV data

Remotely sensed data revealed treatment-level differences in light reflectance and structure (Table 6). Mean NDVI was lower in the BW plots than in the AW or control (*P* < 0.001), and the raw blue light band reflectance was higher in the BW treatment than in either AW or control (*P* < 0.05). The average surface height was lower in the BW than the control treatment (*P* < 0.05); the range and standard deviation of surface heights did not differ across the three treatments. Average green tissue height was lower in the BW treatment than the other treatments (both pairwise comparisons *P* < 0.05), though there was no statistical difference between the AW and control. Finally brown tissue height differed between the control and BW treatments (*P* < 0.05) but neither differed from AW.

**Table 6:** **Comparison of UAV derived variable values by treatment (with ***Typha*** stand as a source of random effects) and Tukey ***post-hoc*** tests (***P*** < 0.05)**.

**Variable**	**Control**		**Below water**	**Above water**
	***df***	**μ ± SE**	**μ ± SE**	**μ ± SE**
NDVI	4	0.27 ± 0.02^a^	0.13 ± 0.02^b^	0.24 ± 0.02^a^
Green cover (%)	4	35.79 ± 12.52^a^	15.84 ± 6.02^b^	22.60 ± 8.06^ab^
Brown cover (%)	4	64.21 ± 12.51	64.04 ± 18.36	76.94 ± 17.91
Water cover (%)	4	0.01 ± 0.00^a^	20.14 ± 3.74^b^	0.48 ± 2.14^a^
Raw blue-band reflectance	4	110.51 ± 4.39^a^	121.23 ± 1.01^b^	110.97 ± 4.40^a^
Surface height (m)	4	0.84 ± 0.04^a^	0.51 ± 0.08^b^	0.71 ± 0.04^ab^
Surface height range (m)	4	0.99 ± 0.18	1.26 ± 0.26	0.88 ± 0.22
Surface height st. dev (m)	4	0.17 ± 0.02	0.16 ± 0.03	0.15 ± 0.03
Green tissue height (m)	4	0.89 ± 0.04^a^	0.51 ± 0.08^b^	0.74 ± 0.05^a^
Brown tissue height (m)	4	0.82 ± 0.03^a^	0.52 ± 0.04^b^	0.70 ± 0.08^ab^

*Significant differences between treatments indicated by non-overlapping superscript letters*.

## Discussion

We evaluated the community composition of young stands of invasive *Typha*, a taxa which invades temperate wetlands in North America (Galatowitsch et al., 1999), experimentally imposed two types of mechanical harvesting treatments (above water harvesting & below water harvesting), assessed the vegetation, structural, and light reflectance values 1-year following treatment, and compared UAV-collected data with field-collected data to evaluate the efficacy of restoration monitoring using remote sensing approaches.

### Experimental evaluation of EDRR restoration strategies

Our results support our hypothesis (1a) that the impacts of invasive plants would be temporally mediated: as predicted, prior to treatment the oldest portions of *Typha* stands had greater *Typha* biomass and cover, greater total detritus, and lower *Carex* spp. cover, Shannon diversity (H′), and species richness than the most recently invaded portions of the wetland. By contrast, plant communities with subdominant *Typha* had greater H′, species richness, and *Carex* spp. cover than the oldest portion of stands. Further, the *Typha* stand-interior subplots differed from the exterior subplots in nearly all measured values (Tables 3, 4). These patterns follow those documented by Mitchell et al. (2011) and Windham and Lathrop (1999) for *T*. × *glauca* and *Phragmites australis* respectively.

Secondly, we predicted that wetland areas with older *Typha* would be more resistant to restoration efforts than areas with younger *Typha* (1b). We found limited evidence to support this hypothesis 1-year post-treatment, with treatment effects largely overwhelming age effects. Interestingly, the AW treatment showed the strongest relationships across subplots: *Carex* spp. cover was more abundant on the *Typha* stand periphery following treatment and there was no measured reduction in *Carex* spp. cover in the youngest portions of *Typha* stands as compared to the control; near the stand center, *Carex* spp. cover approximated the post-treatment cover values of the BW treatments (Figure 3). This pattern indicates that at low levels of *Typha* cover and in young stands of *Typha*, one AW biomass harvest treatment did not negatively impact *Carex* spp. cover. In contrast, glyphosate treatments are apt to reduce not only target invasive species but non-target species, including *Carex* spp. and other native graminoids (Lawrence et al., 2016b). However, at high levels of *Typha* dominance, *Carex* spp. cover was reduced by cutting biomass above water.

We predicted that (2) above-water harvesting would reduce *Typha* dominance, alter the physical structure of the vegetation, and increase native species diversity. This hypothesis proved to be only partially supported, in part because of weaker than expected responses to AW treatment and in part because repeated measures analysis revealed several unanticipated changes in control treatments between sampling years (Table 5), complicating interpretation. For instance, *Typha* cover, standing dead vegetation, and total vegetation cover were reduced 1-year post treatment in both the AW treatments and the control treatments. Furthermore, the AW treatment did not affect *Typha* biomass. The changes associated with control plots between years were likely a result of (a) a sustained increased in Great Lakes water levels over the period of the experiment (Gronewold et al., 2013), as high water levels have been associated with reduced dominance by invasive *Typha* in the upper Great Lakes (Gathman et al., 2005), and (b) phenological variation between years, despite sampling at roughly the same date. In contrast, both H′ and floating and submerged aquatic species cover increased with AW treatment, as compared to the controls. Therefore, the effects of AW treatments on measures of plant community composition and invasive plant dominance were mixed 1-year following treatment (Table 5). We expect that native species may respond more vigorously with additional time following treatment, as a lag in native plant response to invasive plant removal was observed by Lishawa et al. (2015) in northern Michigan coastal wetlands and a similar delayed response is common following water level fluctuations in the Great Lakes (Frieswyk and Zedler, 2007).

We hypothesized that (3) below-water harvesting would more effectively reduce *T*. × *glauca* dominance, but at the cost of also reducing native species diversity. This hypothesis was largely supported; *Typha* cover and biomass were lower in the BW treatments than the controls, as were species richness, H′, *Carex* spp. cover, and graminoid cover (Table 5). BW did have the positive effect of increasing floating and submerged aquatic species cover and decreasing litter and standing dead cover (Figure 3). More broadly, BW cutting shifted the wetland ecosystem from a tall, dense, green, emergent, and invasive *Typha*-dominated community, to a community with more open water and floating and submerged aquatic plant dominance. We found that BW treatments altered a wide suite of measured plant community and structure values as compared to the controls and altered more measured community composition and structure variables than AW treatments; reducing *Typha* cover and biomass, detritus, and standing dead litter, but also reducing species richness and H′ (Figure 3, Table 5). BW plots also had lower average surface elevation and green tissue elevation, lower NDVI, and higher water cover and blue light spectrum reflectance than control or AW treatments, illustrating an increase in open structure and more exposed standing water (Table 6). Aquatic plant species cover was highest in the BW treatments (Figure 3). The submerged aquatic species *Utricularia intermedia* was a significant indicator of BW treatment, likely due to increased openness following removal of vegetation and litter. These vegetation responses were largely unsurprising as cutting *T*. × *glauca* below-water is considered to be an effective method of local eradication (Apfelbaum, 1985), because roots and rhizomes are deprived of oxygen when above-water litter and cut stems have been removed (Sale and Wetzel, 1983; Jordan and Whigham, 1988). The observed reduction in *Carex* spp., graminoid cover, species richness, and species diversity associated with BW treatments illustrates that this management techniques effectively kills both native and non-native emergent macrophytes.

Above water and BW treatments altered plant community composition and structure and in the case of many measured variables, the responses varied significantly from the outer edge of a *Typha* stand to the interior. For instance, *Typha* cover and biomass were significantly higher in both the AW and BW treatments in the stand-interior most subplots (subplot E) as compared to the subplots closest to the stand margin but still within the *Typha* stand (subplot B), in contrast with control plots (Figure 3). This indicates that younger portions of *Typha* stands were more *restorable*, that is treatments were more effective at maintaining low levels of *Typha* dominance. AW treatment reduced *Typha* cover and appeared to favor *Carex* spp. cover in the subdominant and youngest portion of *Typha* stands. This pattern supports the hypotheses that early treatment will be more effective at controlling an invasive plant, and to our surprise indicates that native *Carex* spp. does not appear to be negatively affected by a single aboveground harvest.

### Evaluation of UAV data for post-restoration monitoring

We found that several measures were directly comparable between UAV and ground-measured data types. Particularly useful were the vegetation land cover categories derived from NDVI values (green tissue, brown tissue), which compared well with field measures (total vegetation cover and detritus; Table 6). These measures are critically important response variables to assess the effects of invasive wetland plant management, and UAV data appear to be a suitable proxy for field monitoring data, which are more time intensive.

UAV-derived measures not only confirmed patterns described from the field-derived measures, but exposed additional patterns in structural heterogeneity, which were not effectively measured on the ground. The surface elevation data revealed a reduction in mean vegetation canopy height, brown tissue height, and overall surface height in the BW treatments as compared to the control (Table 6). UAV-derived data are therefore capable of providing additional insights into wetland vegetation structure and complexity.

Some limitations of UAV data replacing field data were apparent, however, in areas with more open water. NDVI values differed widely between UAV and field measures in the BW plots (Figure 4). The NDVI water category was an underestimate of field measures, and the brown vegetation was an overestimate likely because sediment below the shallow-water surface was sometimes classified as brown vegetation and sometimes classified as water. This shows a current limitation in our use of UAV-collected data in wetland ecosystems. Characterizing the degree of open-water in restored wetlands is an important measure of monitoring habitat-value responses, and additional effort is warranted to improve the accuracy of distinguishing shallow water from submerged objects such as aquatic vegetation, leaf litter, and sediment. It may be possible to improve accuracy by using polarized filters, alternative sensors, or flying in different weather conditions to increase the contrast between brown tissue or sediment and shallow water.

### Ecological restoration implications

The results of our study have several implications for wetland restoration, invasive species management, and post-management monitoring. The variable response of *Carex* spp. to harvesting across a *Typha* age and dominance gradient has direct restoration implications. *Carex* spp. are highly important dominant species in wet meadow ecosystems throughout North America and across the globe (Bernard, 1990). Passive restoration efforts (which do not include sowing seeds) have been largely ineffective at enhancing *Carex* spp. dominance (Mulhouse and Galatowitsch, 2003; Hall and Zedler, 2010) due to relatively short term seed viability (van der Valk et al., 1999) and high light requirements for germination (Kettenring et al., 2006). Further, Lishawa et al. (2015) found that in a northern Great Lakes coastal wetland, *Carex* spp. diversity and abundance took 2 years following *Typha* biomass removal to increase from pre-treatment levels. Therefore, we should expect that *Carex* spp. would not respond vigorously from the seed bank 1-year following a passive mechanical treatment and a reduction in water levels would be necessary for widespread *Carex* spp. germination to occur (Keddy and Reznicek, 1986). However, vegetative spread of *Carex* around the margins of a *Typha* stand could increase the extent of wet meadow natural communities, as Hall and Zedler (2010) also concluded. If *Carex* species are more resilient than *Typha* in recently-established *Typha* stands, repeated above-water harvesting may work to push the invasion front back and re-establish *Carex* spp. dominance. Repeated subsequent harvests along the margin of the *Carex* spp./*Typha* dominated zone would further elucidate the effectiveness of this approach. The contrasting responses of *Carex* spp. following treatment indicate that stand-age should be considered when conducting invasive plant treatments, and care should be used to minimize harming native *Carex* spp. plants when they are growing within stands of invasive *Typha*. Furthermore, planting native graminoids following *Typha* treatment may be necessary in more advanced stages of invasion (Hall and Zedler, 2010).

Our results point to the effectiveness of non-chemical management in young stands to control invasive *Typha*. Particularly, we found that the use of harvesting followed by below water cutting in areas with high levels of *Typha* dominance was effective at controlling *Typha*, creating more open water habitat, and facilitating increased coverage of native floating and submerged aquatic plants, but these advantages came with the cost of decreasing native species diversity. In mixed stands of *Carex* spp. and *Typha* and in the youngest portions of *Typha* stands, it may be more appropriate to cut all vegetation above water, as that treatment appears to reduce *Typha* dominance, while maintaining graminoid and *Carex* spp. cover.

The differing responses we observed between AW and BW cutting suggest that an integrated management approach that takes into account the age of invasive plant populations and existing plant community composition will be the most effective strategy for managing *Typha* invasions in high-quality wetlands. In well-established *Typha* stands that lack remnant *Carex* spp. populations, BW cutting will increase interspersion of open water that can be used as stopover and breeding habitat for migratory waterfowl, secretive marsh birds, and shorebirds (Murkin et al., 1982; Rehm and Baldassarre, 2007; Darrah and Krementz, 2010; Webb et al., 2010) and juvenile fish. A single AW cutting in these well-established stands will not substantially decrease the density of living *Typha* stems, but it will remove accumulated leaf litter, likely making the habitat more accessible to wading birds, fish, and amphibians. In recently established stands, however, AW cutting will likely suppress the *Typha* invasion and promote *Carex* spp. dominance.

## Conclusions

Our findings suggest that mechanically harvesting the above-water biomass of young stands of invasive *Typha* and harvesting older stands below-water will promote native community resilience, and increase floating and submerged aquatic species abundance, which are some of the most vulnerable wetland plant guilds to plant invasions (Stiers et al., 2011). These results add further evidence that mechanical treatment and biomass harvest are effective alternatives to *Typha* management approaches that rely on herbicide or fire. Scientifically vetted management techniques are increasingly important in light of the continued expansion of *Typha* into high-quality Great Lakes coastal wetlands (Lishawa et al., 2010), and predicted climate change driven reductions in water levels (Angel and Kunkel, 2010), which will favor *Typha* into the foreseeable future.

UAVs provided quickly collected data compared to field monitoring, and effectively measured plant cover and vegetation structural responses to different treatments. The results of our UAV derived NDVI and surface elevation model analyses demonstrate that open water and vegetative structural complexity, two important fish and wildlife metrics, can be quantified using UAV-collected imagery. Because the results of our UAV data analysis were largely corroborated by on-the-ground vegetative monitoring, we believe that drone-collected data have the potential to be used as a spatially appropriate method to supplement and enhance wetland restoration monitoring. However, the level of detail provided by field data collection, particularly in evaluating biodiversity, cannot be replicated with remotely sensed data. Therefore, we suggest pairing UAV flights with targeted high-intensity field data collection to maximize the quality of post-restoration vegetation monitoring.

## Author contributions

SL, BC, NR, JB, EC, and DA conceived the idea; SL, BC, and NR designed the experiment. JT and JB provided remote sensing expertise, SL and UAV crew collected UAV data, and JT conducted UAV data post-processing. SL, DC, JB, AM, and JL led field crews and collected field data. SL led all data analysis and manuscript writing. All authors edited the manuscript.

## Funding

Funding to support this work came from US Environmental Protection Agency GLRI grant GL-00E1293, State of Michigan DNR MISGP grant IS15-2003, a Loyola University Chicago Office of the Provost award to Lishawa, and a Bureau of Indian Affairs award to the Sault Ste. Marie Tribe of Chippewa Indians.

### Conflict of interest statement

The authors declare that the research was conducted in the absence of any commercial or financial relationships that could be construed as a potential conflict of interest.
